# Differential Nitrogen Nutrition Modifies Polyamines and the Amino-Acid Profile of Sweet Pepper Under Salinity Stress

**DOI:** 10.3389/fpls.2019.00301

**Published:** 2019-04-02

**Authors:** M. C. Piñero, Manuel E. Porras, Josefa López-Marín, Mari C. Sánchez-Guerrero, Evangelina Medrano, Pilar Lorenzo, Francisco M. del Amor

**Affiliations:** ^1^Department of Crop Production and Agri-Tecnology, Murcia Institute of Agri-Food Research and Development, Murcia, Spain; ^2^Agricultural Research and Development Centre of Almería (IFAPA), Almería, Spain

**Keywords:** nitrogen, salinity, *Capsicum annuum* L., nutrients, free amino acids, polyamines

## Abstract

The horticultural industry demands high-quality resources to achieve excellence in yield and optimal revenues. Nitrogen is a pivotal nutrient to accomplish these goals for plant growth and product quality. However, competition for water in semi-arid regions can force the use of brackish waters, which can impair N uptake. The lower N uptake can be due to several reasons, such as an antagonism between ions, an absence of ATP, and/or alteration of N metabolism. The effect of supplying N as ${\text{NO}}_{3}^{-}$ alone or in combination with ${\text{NH}}_{4}^{+}$, coupled with low or high salinity (8 or 20 mM NaCl), has been studied in sweet pepper fruits (*Capsicum annuum* L. cv. Melchor). The application of ${\text{NH}}_{4}^{+}$ at high salinity affected chromatic parameters (a^∗^, b^∗^, and C^∗^), while chlorophyll a and b levels declined and β-carotene increased. The concentrations of P, K, Ca, Mg, and Cu were reduced in the fruits of plants irrigated with ${\text{NH}}_{4}^{+}$. The concentration of Na was only reduced when ${\text{NH}}_{4}^{+}$ was supplied. Likewise, the concentration of total phenolics was also reduced at high salinity. However, total protein was unaffected. The amino acid profile was altered by the supply of ${\text{NH}}_{4}^{+}$, which reduced the concentrations of histidine and phenylalanine. Moreover, the concentrations of putrescine and cadaverine were increased by ${\text{NH}}_{4}^{+}$ at high salinity, whereas that of cadaverine was reduced by ${\text{NH}}_{4}^{+}$ at low salinity. The observed changes in fruit quality triggered by salinity, under the conditions of this study, should be borne in mind for this crop with regard to the envisaged palliative effect of the supply of N-${\text{NH}}_{4}^{+}$.

## Introduction

Consumers are increasingly demanding high-quality products with benefits for human health (Bortolin et al., 2016). However, the agricultural industry usually prioritizes the maximization of yield and quality without investing in the improvement of plant tolerance of potentially harmful abiotic stress factors, which frequently impair crop yields. The current climate drift that presumably will increase the frequency and severity of dry heatwaves is now a source of concern to many growers worldwide, but especially in Mediterranean-climate areas (Surasinghe, 2011). Therefore, new, efficient, and effective ameliorative tools are necessary, to reduce the impacts and to maintain ecosystem biodiversity, sustainability, economic growth, food security, and social equity (Dhankher and Foyer, 2018). The competition for good-quality water, which is forcing farmers to use brackish waters for irrigation, could be alleviated by the use of salt-tolerant crops or appropriate management (fertirrigation) practices (Piñero et al., 2018). Salinity is one of the main stressors limiting plant development and crop productivity. Munns and Tester (2008) observed that under salinity stress the plant N content declines while the leaf Cl^−^ content increases. However, the negative effects of salinity could, in part, be compensated by an appropriate, balanced N supply. Some studies indicated that the supply of ${\text{NO}}_{3}^{-}$ as the sole N source may be detrimental under salt stress, since salinity reduces its uptake rate (Kant et al., 2007) and decreases the plant N content (Piñero et al., 2016). Several reasons have been put forward for the decrease in leaf N content under salinity: (i) an antagonism between Cl^−^ and ${\text{NO}}_{3}^{-}$ transport and/or inactivation of ${\text{NO}}_{3}^{-}$ transporters by the toxic effects of salinity; (ii) an absence of ATP, which is required for active ${\text{NO}}_{3}^{-}$ transport (Piñero et al., 2016); and (iii) an alteration in the activities of enzymes involved in N metabolism induced by salt (Srivastava and Mishra, 2014). The supply of ${\text{NH}}_{4}^{+}$ has an advantage, related to the lower energy cost of its uptake, compared with ${\text{NO}}_{3}^{-}$ (Fernández-Crespo et al., 2012), but it can induce cell acidification, nutrient deficiencies, and inhibition of root growth (Sarasketa et al., 2016). Moreover, ${\text{NH}}_{4}^{+}$ has also been associated with amino acids that can act as signaling molecules to trigger metabolic pathways that could limit oxidative damage (Hessini et al., 2013), such as that provoked by salinity stress. Consequently, the growth response of plants to N fertilization under salinity stress varies depending on whether N is supplied as ${\text{NO}}_{3}^{-}$ or ${\text{NH}}_{4}^{+}$, as well as on the species considered (Misra and Dwivedi, 1990).

Sweet pepper (*Capsicum annuum* L.) is one of the most valuable crops in the Mediterranean area and is usually cultivated in greenhouses, which allow higher yield and exceptional fruit quality in comparison with open field (conventional cultivation) conditions (Serret et al., 2018). The fruit contains a large number of health-promoting compounds such as vitamins, carotenoids, lycopene, capsaicinoids, phenolic compounds, and amino acids, all of which have antioxidant properties and provide protection against cancer (Bramley, 2000; Navarro et al., 2006; González-Chavira et al., 2018). Moreover, the synthesis of phenolic compounds, vitamin C, and carotenoids in pepper and other vegetables depends on several factors, such as the cultivar, agricultural practices, maturity, and storage conditions. It has been observed that the availability of N has the ability to modify the synthesis of phenolic compounds and soluble solids (Doll et al., 1994; González-Chavira et al., 2018).

Polyamines, also known as biogenic amines, are low molecular weight compounds involved in plant responses to abiotic and biotic stresses (Hussain et al., 2011), as well as in the processes of cell growth and differentiation (Regla-Márquez et al., 2016). They are also linked to molecular plant defense mechanisms involved in the scavenging and/or generation of free radicals, regulation of gene expression, and formation of toxic defense products (Cai et al., 2015). Moreover, polyamines are also related to the hormonal balance (Houdusse et al., 2008) and the direct effect of climate change, as influenced by CO_2_ concentrations (Piñero et al., 2017).

In this study, we hypothesized that the additional supply of ${\text{NH}}_{4}^{+}$ to the nutrient solution (instead of using ${\text{NO}}_{3}^{-}$ as the sole N source) might partially or totally overcome the predicted deleterious effect of salinity on the fruit quality of sweet pepper, as this crop is considered salt sensitive (del Amor and Cuadra-Crespo, 2011; Piñero et al., 2014). Most plants show a strong preference for ${\text{NO}}_{3}^{-}$ over ${\text{NH}}_{4}^{+}$ ions, and others grow best if they have access to both ${\text{NO}}_{3}^{-}$ and ${\text{NH}}_{4}^{+}$ (Zhou et al., 2011). However, the optimum ${\text{NO}}_{3}^{-}$ /${\text{NH}}_{4}^{+}$ ratio changes depending on the plant species, the stage of development, and the environmental conditions (Hu et al., 2015). Since the reported studies concerning the effect of different N forms and salinity on sweet pepper quality are very few, this study offers a new insight into the impact of the interaction between the ${\text{NO}}_{3}^{-}$ /${\text{NH}}_{4}^{+}$ ratio and salinity, in this important crop. Therefore, our objective was to determine the influence of the ${\text{NO}}_{3}^{-}$ /${\text{NH}}_{4}^{+}$ nutritional regime, under salt stress, on the fruit colorimetric properties, pigments (chlorophylls, lycopene, and β-carotene), mineral composition, total protein and total phenolics contents, polyamines, and amino-acids profile.

## Materials and Methods

### Plant Material and Growth Conditions

The experiments took place in a greenhouse located in the IFAPA center “La Mojonera” (Almería, Spain, latitude 36°48′ N, longitude 2°41′ W). It was of the multi-tunnel type (3 spans), oriented east–west, and had an area of 720 m^2^. The greenhouse height was 4.7 m to the ridge and 3 m to the gutter of each span. The greenhouse cover was thermal polyethylene (PE) (0.2 mm thick). Ventilation was provided by two roof vents (opening area: 1 m × 30 m) and two side vents (opening area: 1.5 m × 26 m) set in both the south and north arches. The greenhouse was equipped with a commercial climate control system (CDC, INTA S.A.). A continuous register of the temperature and humidity (HMP45C sensors, Campbell Sci.) was maintained. In the greenhouse, pepper seedlings (cv. Melchor) were transplanted on 19 August 2013, two plants into each 27-L container filled with perlite, with a density of 2.5 plants per m^2^. The cultivation was carried out with a type of Dutch pruning; two stalks were left on each plant.

Water and fertilizer were delivered by an automated drip irrigation system (CDN, INTA S.A.). The nutrient solution was applied with one dripper (3 L h^−1^) per container. Two levels of nutrient solution salinity were used: 8 mM NaCl (C) for half of the plants and 25 mM NaCl (S) for the other half. Also, the plants in each salinity treatment were supplied with different N sources: half received ${\text{NO}}_{3}^{-}$ alone and the other half a ${\text{NO}}_{3}^{-}$ /${\text{NH}}_{4}^{+}$ mixture. To adjust the N input to the crop demand, two phases were established. In the first phase (until October 31) the N inputs were: ${\text{NO}}_{3}^{-}$ 12 mM ${\text{NO}}_{3}^{-}$ and ${\text{NO}}_{3}^{-}$ /${\text{NH}}_{4}^{+}$ 10 mM ${\text{NO}}_{3}^{-}$ + 2 mM ${\text{NH}}_{4}^{+}$; in the second phase (October 31 to the end of the cycle) the contributions were: ${\text{NO}}_{3}^{-}$ 10 mM ${\text{NO}}_{3}^{-}$ and ${\text{NO}}_{3}^{-}$ /${\text{NH}}_{4}^{+}$ 8 mM ${\text{NO}}_{3}^{-}$ + 2 mM ${\text{NH}}_{4}^{+}$. The volume of nutrient solution supplied by each irrigation event was 500 mL per container. The irrigation frequency fluctuated between one and five times per day depending on the needs of the plants, maintaining approximately 40% drainage. The harvest period was between 28 October 2013 and 24 February 2014, the fruits being harvested once they had reached commercial maturity (red color). At the second harvest (29 January 2014) 12 fruits per treatment were collected from different plants, for the measurement of color, chlorophylls, lycopene, β-carotene, mineral content, total proteins, total phenolic compounds, amino acids, and polyamines. Two fruits were considered a sample; therefore, the analyses were carried out using six replicates per treatment.

### Skin Color

Pepper fruit color was measured with a Konica-Minolta CR-300 colorimeter (Konica-Minolta, Kyoto, Japan) having a D65 illuminant, making three measurements along the equatorial perimeter. The color data are provided as CIEL^∗^*a^∗^b*^∗^ coordinates, which define the color in a three-dimensional space. *C*^∗^ is chroma [*C*^∗^ = √(*a*^∗2^) + (*b*^∗2^)], 0 being at the center of a color sphere, and the value increases according to the distance from the center. Finally, *h_ab_* is the hue angle [*h_ab_* = *arc tg*(*b*^∗^*a*^∗^)], which is defined as starting at the +*a*^∗^ axis and is expressed in degrees; 0° would be +*a*^∗^ (red), 90° would be +*b*^∗^ (yellow), 180° would be −*a*^∗^ (green), and 270° would be −*b*^∗^ (blue).

### Fruit Chlorophylls, Lycopene, and β-Carotene

The β-carotene, lycopene, and chlorophylls were extracted from 1-g samples of frozen pepper fruits (−80°C) with 25 mL of acetone–hexane (2:3) solvent. The samples were homogenized using a polytron and centrifuged at 3,500 rpm for 6 min, at 4°C. Subsequently, the optical density of the supernatant was measured spectrophotometrically at wavelengths of 663, 645, 505, and 453 nm. The concentrations of chlorophylls *a* and *b*, lycopene, and β-carotene were calculated using Nagata and Yamashita (1992) equations:

$$\text{Chlorophyll }a\;(\text{mg}100{\text{ mL}}^{-1})=0.999^{*}{\text{A}}_{663}--0.0989^{*}{\text{A}}_{645}.$$

$$\text{Chlorophyll }b\;(\text{mg}100{\text{ mL}}^{-1})=-0.328^{*}{\text{A}}_{663}+1.77^{*}{\text{A}}_{645}.$$

$$\text{Lycopene }(\text{mg}100{\text{ mL}}^{-1})=-0.0458^{*}{\text{A}}_{663}+0.204^{*}{\text{A}}_{645}+0.372^{*}{\text{A}}_{505}-0.0806^{*}{\text{ A}}_{453}.$$

$$\text{β-Carotene }(\text{mg}100{\text{ mL}}^{-1})=0.216^{*}{\text{A}}_{663}-1.22^{*}{\text{A}}_{645}-0.304^{*}{\text{A}}_{505}+0.452^{*}{\text{ A}}_{453}.$$

### Mineral Content

The Ca, K, Mg, B, Cu, Fe, Mn, P, and Na concentrations in the dry matter of fruits were determined with an inductively-coupled plasma (ICP) spectrometer (Varian Vista MPX, Palo Alto, CA, United States). An ETHOS ONE microwave digestion system (Milestone, Inc., Shelton, CT, United States) was applied for fruit sample preparation. This digestion procedure has many advantages due to the speed of the digestion process, the lower acid consumption and lower possibility of contamination, and the high extraction efficiencies (Soylak et al., 2004).

### Total Protein

The fruit dry weight was determined after at least 72 h at 70°C and the total nitrogen was measured in the dry matter, using a LECO FP-528 (Leco Corporation, St. Joseph, MI, United States). We use the conversion factor 6.25 to convert total nitrogen to total protein (Jones et al., 1942).

### Total Phenolic Compounds

The total phenolic compounds were extracted from 0.5 g of frozen pepper fruit (−80°C) with 5 mL of 80% acetone. The homogenate was centrifuged at 10,000 rpm for 10 min, at 4°C. For the determination, Folin–Ciocalteu reagent was used, diluted with Milli-Q water (1:10). The diluted reagent (1 mL) was mixed with 100 μL of supernatant and 2 mL of Milli-Q water, and 5 mL of sodium carbonate (20%) were then added. The mixture was kept for 30 min in the dark and then the absorbance was measured at 765 nm, according to the methodology of Kähkönen et al. (1999). The total phenolic content was expressed as gallic acid equivalents, in mg g^−1^ fresh weight.

### Free Amino Acids

The free amino acids were extracted from fruits frozen at −80°C: the homogenate was extracted, after vortexing at 5,000 rpm (10 min, 4°C), and analyzed by the AccQ⋅Tag-ultra ultra-performance liquid chromatography (UPLC) method (Waters, UPLC Amino Acid Analysis Solution, 2006). For derivatization, 70 μL of borate buffer were added to the hydrolyzed sample or to 10 μL of the fruit homogenate. Next, 20 μL of reagent solution were added. The reaction mixture was mixed immediately and heated at 55°C for 10 min. After cooling, an aliquot of the reaction mixture was used for UPLC injection. The UPLC was performed with an Acquity system (Waters, Milford, MA, United States) equipped with a fluorescence detection (FLR) system. A BEH C18 100 mm × 2.1 mm, 1.7 μm column (Waters) was used. The flow rate was 0.7 mL min^−1^ and the column temperature was kept at 55°C. The injection volume was 1 μL. The excitation (λex) and emission (λem) wavelengths were set at 266 and 473 nm, respectively. The solvent system consisted of two eluents: (A) AccQ⋅Tag-ultra eluent A concentrate (5%, v/v) and water (95%, v/v); (B) AccQ⋅Tag-ultra eluent B. The following elution gradient was used: 0–0.54 min, 99.9% A–0.1% B; 5.74 min, 90.9% A–9.1% B; 7.74 min, 78.8% A–21.2% B; 8.04 min, 40.4% A–59.6% B; 8.05–8.64 min, 10% A–90% B; 8.73–10 min, 99.9% A–0.1% B. Empower 2 (Waters) software was used for system control and data acquisition. External standards (Thermo Scientific) were used for quantification of (Ala) alanine; (Arg) arginine; (Asp) aspartic acid; (Cys) cysteine; (Glu) glutamic acid; (Gly) glycine; (His) histidine; (Ile) isoleucine; (Leu) leucine; (Lys) lysine; (Met) methionine; (Phe) phenylalanine; (Pro) proline; (Ser) serine; (Thr) threonine; (Tyr) tyrosine; and (Val) valine.

### Polyamine Analysis

Free polyamines were extracted by homogenizing 1.0 g of tissue in 10 mL of 5% perchloric acid, using a Polytron (Kinematica, Bohemia, NY, United States) homogenizer, and were analyzed by the benzoylation method (Serrano et al., 1995), using high-performance liquid chromatography (HPLC) (Hewlett-Packard). As an internal standard, 1,6-hexanediamine ([100 nmol (g fresh weight)^−1^ of tissue] was used, and standard curves of putrescine, cadaverine, and histamine were prepared. The results are expressed as nmol (g fresh weight)^−1^.

### Statistical Analysis

The data were tested first for homogeneity of variance and normality of distribution. The significance was determined by analysis of variance (ANOVA) and the significance (*P* ≤ 0.05) of differences between mean values was tested by Duncan’s New Multiple Range Test, using Statgraphics Centurion^®^ XVI (StatPoint Technologies, Inc.). Four combinations of treatments were used, involving two N forms (${\text{NO}}_{3}^{-}$ or ${\text{NO}}_{3}^{-}$ /${\text{NH}}_{4}^{+}$) and two levels of nutrient solution salinity (8 and 25 mM NaCl), with six replications per combination.

## Results and Discussion

### Color, Chlorophylls, Lycopene, and β-Carotene

**FIGURE 1:**
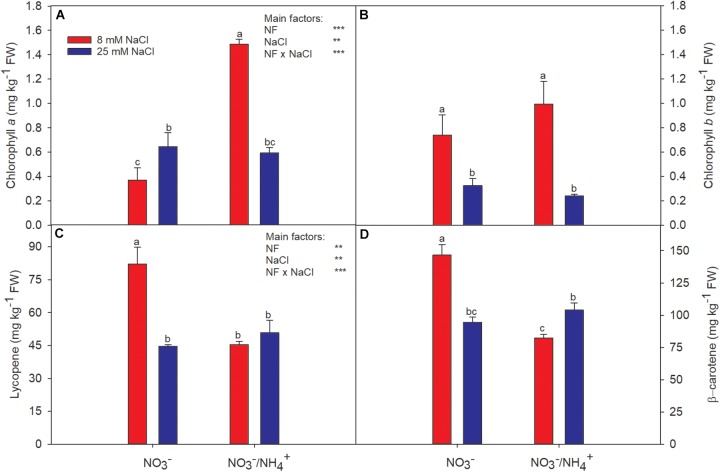
Effect of two different N forms (NF) (${\text{NO}}_{3}^{-}$ and ${\text{NO}}_{3}^{-}$ /${\text{NH}}_{4}^{+}$) and salinity on sweet pepper fruits: **(A)** chlorophyll *a*, **(B)** chlorophyll *b*, **(C)** lycopene, and **(D)** β-carotene. Values with the same letter are not significantly different at *P* ≤ 0.05. Analysis of variance: ^∗^*P* ≤ 0.05; ^∗∗^*P* ≤ 0.01; ^∗∗∗^*P* ≤ 0.001.

The fruit skin lightness (L^∗^) values were only affected by salinity. However, the fruit of plants irrigated with ${\text{NH}}_{4}^{+}$-containing nutrient solution, averaged over the two salinity levels, had lower values of the parameters a^∗^, b^∗^, and C^∗^, compared with the supply of ${\text{NO}}_{3}^{-}$ alone. Fruits of plants grown with ${\text{NH}}_{4}^{+}$ were less red in color (*a*^∗^ = 24.01 and *b*^∗^ = 11.56) and had a less intense and less vivid color (*C*^∗^ = 26.73), although such differences would hardly be perceived by consumers (Table 1). Additionally, the lower a^∗^ values coincided with a higher chlorophyll *a* concentration and lower contents of lycopene and β-carotene (Figure 1). Thus, this reduction in the intensity of the red color appears to be due to reduced chlorophyll degradation and a considerable reduction in the contents of lycopene and β-carotene. On the other hand, the salt treatment had no significant general effect on the CIELab color coordinates (with the exception of L^∗^). This could be because the chlorophyll *a* increased in the same proportion (from 0.37 to 0.65 mg kg^−1^ FW) as the degradation of chlorophyll *b* (from 0.74 to 0.32) (Figure 1A,B). Salinity significantly modified the lycopene and β-carotene contents in fruits (Figure 1C,D). The concentrations of lycopene and β-carotene were reduced at high salinity in fruits grown with ${\text{NO}}_{3}^{-}$ alone. But, this effect was not observed when ${\text{NH}}_{4}^{+}$ was added to the nutrient solution, especially for β-carotene – whose concentration was significantly increased. These responses highlight the interaction between the N form and salinity concerning chlorophyll a, lycopene, and β-carotene, while chlorophyll b was only clearly affected by salinity.

**Table 1:** Effect of two different N forms (NF) (${\text{NO}}_{3}^{-}$ and ${\text{NO}}_{3}^{-}$ /${\text{NH}}_{4}^{+}$) and salinity (8 and 25 mM NaCl) on sweet pepper fruits: CIEL^∗^a^∗^b^∗^ color coordinates.

Nitrogen form	[NaCl] mM	*L*^∗^	*A*^∗^	*b*^∗^	*C*^∗^	h_ab_
${\text{NO}}_{3}^{-}$	**8**	31.75 ± 0.13 ab	26.43 ± 0.49 b	12.77 ± 0.22 b	29.48 ± 0.53 c	26.12 ± 0.41 ab
	**25**	32.09 ± 0.28 b	25.31 ± 0.37 ab	12.89 ± 0.25 b	28.40 ± 0.39 bc	26.92 ± 0.49 b
${\text{NO}}_{3}^{-}$ /${\text{NH}}_{4}^{+}$	**8**	31.25 ± 0.32 a	24.01 ± 0.59 a	11.56 ± 0.32 a	26.73 ± 0.64 a	26.06 ± 0.20 ab
	**25**	32.12 ± 0.29 b	24.68 ± 0.72 a	12.29 ± 0.16 b	27.59 ± 0.52 ab	25.65 ± 0.13 a
ANOVA^a^						
N form		ns	^∗^	^∗∗^	^∗∗^	ns
NaCl		^∗^	Ns	ns	ns	ns
NF^b^ × NaCl		ns	Ns	ns	ns	ns

*Different letters within a column indicate significant (P ≤ 0.05) differences between treatments. ^a^Analysis of variance: ns, not significant; ^∗^P ≤ 0.05; ^∗∗^P ≤ 0.001. ^b^Nitrogen form.*

### Mineral Content

The P, K, Ca, Mg, and Cu concentrations in pepper fruits were influenced significantly by the salinity and N form, decreasing with the presence of ${\text{NH}}_{4}^{+}$ in the nutrient solution (Table 2). Thus, the N form used for fertilization can be considered an important factor in the uptake of mineral nutrients by plants (Kowalska and Sady, 2012). In plants, ${\text{NH}}_{4}^{+}$ can act through different physiological and biochemical mechanisms – like acidification of the growth medium and ${\text{NH}}_{4}^{+}$-toxicity *per se*, leading to antagonism in cation uptake, and/or alterations in the osmotic balance (Horchani et al., 2010). Borgognone et al. (2013) studied the mineral composition of tomato and showed that the uptake of K, Ca, and Mg was lower when the proportion of ${\text{NH}}_{4}^{+}$ in the nutrient solution was increased. These authors considered that this was due to the mechanism of charge balance in ion uptake, since N is a dominant macronutrient and its ionic form controls cation and anion uptake. Horchani et al. (2010) observed that ${\text{NH}}_{4}^{+}$ toxicity led to antagonism in cation uptake and/or alterations in the osmotic balance, which lowered the uptake of cations. However, in the current work, the rise in the external salinity only had a notable effect on the concentration of Na, which increased from 64 to 322 mg kg^−1^ DW and from 21 to 145 mg kg^−1^ DW in fruits of plants irrigated with ${\text{NO}}_{3}^{-}$ or ${\text{NO}}_{3}^{-}$ /${\text{NH}}_{4}^{+}$, respectively. This agrees with de Souza Miranda et al. (2016), who found that, under salinity, Na^+^ accumulation was severely limited in the presence of ${\text{NH}}_{4}^{+}$. Likewise, Houdusse et al. (2008) pointed out that K and Ca levels declined in plants grown under salinity when they were supplied with ${\text{NH}}_{4}^{+}$. This agrees with our data for fruits and again emphasizes the effect of adding ${\text{NH}}_{4}^{+}$ under saline stress conditions. Moreover, the concentrations of microelements (B, Mn, Fe, and Cu) were not affected by salinity when ${\text{NH}}_{4}^{+}$ was included in the nutrient solution, but they were increased when salinity was imposed without it. Especially notable was the effect of the N form on Cu, since ${\text{NH}}_{4}^{+}$ dramatically reduced the Cu concentration in fruits.

**Table 2:** Effect of two different N forms (NF) (${\text{NO}}_{3}^{-}$ and ${\text{NO}}_{3}^{-}$ /${\text{NH}}_{4}^{+}$) and salinity (8 and 25 mM NaCl) on sweet pepper fruits: mineral contents (on a dry weight basis).

Nitrogen form	[NaCl] mM	P (mg kg^−1^)	K (mg kg^−1^)	Ca (mg kg^−1^)	Mg (mg kg^−1^)	B (mg kg^−1^)	Mn (mg kg^−1^)	Fe (mg kg^−1^)	Na (mg kg^−1^)	Cu (mg kg^−1^)
${\text{NO}}_{3}^{-}$	**8**	2263 ± 33.4 c	22984 ± 817 b	411.4 ± 18.2 b	1361 ± 17 b	9.96 ± 0.3 b	10.13 ± 0.6 a	26.17 ± 0.7 a	64.63 ± 9.2 ab	2.77 ± 0.17 b
	**25**	2124 ± 53.5 bc	22321 ± 563 b	384.3 ± 10.2 b	1348 ± 22 b	11.10 ± 0.4 c	13.16 ± 0.2 b	28.48 ± 0.5 b	322 ± 62.1 c	4.41 ± 0.10 c
${\text{NO}}_{3}^{-}$ /${\text{NH}}_{4}^{+}$	**8**	1807 ± 49.4 a	19751 ± 281 a	10.22 ± 10.2 a	989 ± 44 a	10.48 ± 0.2 bc	8.86 ± 0.4 a	26.19 ± 0.2 a	21 ± 4 a	0.08 ± 0.07 a
	**25**	2026 ± 71.3 b	19419 ± 343 a	14.01 ± 8.9 a	933 ± 41 a	7.66 ± 0.2 a	9.04 ± 0.5 a	25.52 ± 0.2 a	145 ± 26 b	0.13 ± 0.13 a
ANOVA^a^										
N form		^∗∗∗^	^∗∗∗^	^∗∗∗^	^∗∗∗^	^∗∗∗^	^∗∗∗^	^∗∗^	^∗∗^	^∗∗∗^
NaCl		ns	ns	ns	ns	^∗^	^∗∗^	ns	^∗∗∗^	^∗∗∗^
NF^b^ × NaCl		^∗∗^	ns	ns	ns	^∗∗∗^	^∗∗^	^∗∗^	ns	^∗∗∗^

*Different letters within a column indicate significant (P ≤ 0.05) differences between treatments. ^a^Analysis of variance: ns, not significant; ^∗^P ≤ 0.05; ^∗∗^P ≤ 0.01; ^∗∗∗^P ≤ 0.001. ^b^Nitrogen form.*

### Total Protein

**FIGURE 2:**
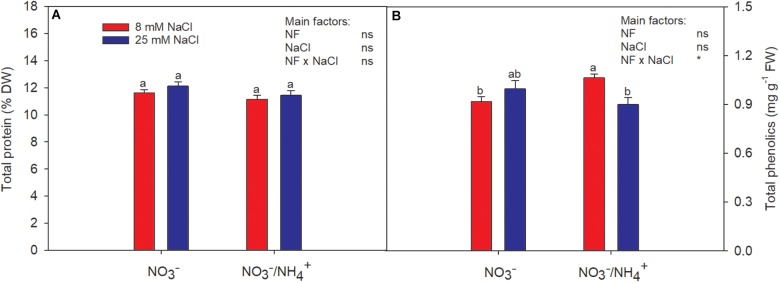
Effect of two different N forms (NF) (${\text{NO}}_{3}^{-}$ and ${\text{NO}}_{3}^{-}$ /${\text{NH}}_{4}^{+}$) and salinity on sweet pepper fruits: **(A)** total protein and **(B)** total phenolics concentration. Values with the same letter are not significantly different at *P* ≤ 0.05. Analysis of variance: ns, not significant; ^∗^*P* ≤ 0.05.

When ${\text{NH}}_{4}^{+}$ was supplied in the nutrient solution, the total protein content was reduced slightly, but did not differ significantly from the levels of the control plants (Figure 2A). Likewise, salinity had no effect on the total protein content.

### Total Phenolics

The content of total phenolics was highest in fruits of plants irrigated with ${\text{NH}}_{4}^{+}$ in the absence of salinity stress (Figure 2B). The total phenolic concentration was increased by 20% in the fruits of sweet pepper by the supply of ${\text{NH}}_{4}^{+}$ in the nutrient solution, compared to the control (Figure 2B), in accordance with Leja et al. (2008). Abu-Zahra (2011) attributed differences in the concentrations of phenolics to nutrient availability. The importance of phenolic compounds lies in the nutritional, organoleptic, and commercial properties of agricultural food products, since they contribute to sensory properties such as color and flavor (Pérez-López et al., 2007). Phenolic compounds in our diet provide health benefits associated with a reduced risk of chronic diseases. It has been found that they have the ability to protect against cardiovascular disease and have anticarcinogenic properties due to their antioxidant activity and their role as free radical scavengers (Gani et al., 2012). Moreover, it is well-known that environmental stresses stimulate the biosynthesis of phenolic compounds (Mitchell et al., 2007). Authors like Wahid and Ghazanfar (2006) found that greater synthesis of phenolics compounds is directly correlated with salt tolerance. However, our study clearly shows that the response to salinity differed according to the N form(s) supplied, being reduced when ${\text{NH}}_{4}^{+}$ was added to the nutrient solution. Therefore, the beneficial effect for human well-being triggered by salinity (referred to earlier) is diminished under such a plant nutrition strategy.

### Free Amino Acids

Amino acids are essential for human health. They are required for the growth, development, regeneration, and reconstruction of the body and are responsible for the production of antibodies, blood cells, hormones, and enzymes (Zou et al., 2015). The concentration of total amino acids was highest in the fruits of the salinity-stressed plants that received ${\text{NH}}_{4}^{+}$ (Table 3). In a more detailed analysis considering each amino acid individually, Gly was the most abundant free amino acid in the fruits of all treatments, and its relative content was not significantly affected by the N form or salinity. Moreover, Pro, Asp, and Ser, which are non-essential amino acids, followed Gly in abundance, in red fruits. The Ser concentration was significantly increased by the interaction between salinity and the supply of ${\text{NH}}_{4}^{+}$. By contrast, the supply of ${\text{NH}}_{4}^{+}$ caused the greatest reduction in the concentrations of Tyr and His (64 and 52%, respectively), while the levels of Tyr (38%) and Met (36%) were also reduced by salinity. Thus, an interaction occurred, since the presence of ${\text{NH}}_{4}^{+}$ in the nutrient solution only resulted in decreased Tyr concentrations under low salinity. Furthermore, salinity did not reduce the Met concentrations when ${\text{NH}}_{4}^{+}$ was added to the nutrient solution.

**Table 3:** Effect of two different N forms (NF) (${\text{NO}}_{3}^{-}$ and ${\text{NO}}_{3}^{-}$ /${\text{NH}}_{4}^{+}$) and salinity (8 and 25 mM NaCl) on sweet pepper fruits: amino acid profiles.

Amino acids (mg/L)	N forms				ANOVA		
	${\text{NO}}_{3}^{-}$		${\text{NO}}_{3}^{-}$ /${\text{NH}}_{4}^{+}$				
	8 mM NaCl	25 mM NaCl	8 mM NaCl	25 mM NaCl	N form	NaCl	NF × NaCl
His	51.1 ± 1.1^b^	49.8 ± 3.4^b^	24.6 ± 2.4^a^	32.2 ± 4.2^a^	^∗∗∗^	ns	ns
Ser	262 ± 7.3^a^	288 ± 7.9^a^	220 ± 9^a^	424 ± 45^b^	ns	^∗∗^	^∗^
Gly	1417 ± 37^a^	1370 ± 75^a^	1325 ± 35^a^	1577 ± 178^a^	ns	ns	ns
Arg	74.5 ± 2.1^b^	64.8 ± 2.0^ab^	57.6 ± 2.0^a^	65.3 ± 5.1^ab^	^∗^	ns	^∗^
Asp	302 ± 14^a^	294 ± 4.6^a^	246 ± 17^a^	314 ± 38^a^	ns	ns	ns
Glu	111 ± 7.3^b^	100 ± 1.1^ab^	90.2 ± 6.8^a^	110 ± 5.7^b^	ns	ns	^∗^
Thr	236 ± 6.8^a^	204 ± 6.8^ab^	176 ± 11^a^	224 ± 22^b^	ns	ns	^∗^
Ala	207 ± 3.0^b^	191 ± 6.1^ab^	158 ± 7.0^a^	222 ± 20^b^	ns	ns	^∗^
Pro	352 ± 16^a^	249 ± 23^a^	267 ± 9.3^a^	355 ± 75^a^	ns	ns	ns
Cys	118 ± 11^b^	103 ± 3.9^ab^	82.9 ± 5.3^a^	114 ± 8.6^b^	ns	ns	^∗^
Lys	108 ± 1.4^b^	88.0 ± 3.3^ab^	70.4 ± 0.8^a^	110 ± 14^b^	ns	ns	^∗^
Tyr	59.8 ± 2.8^c^	37.0 ± 2.1^b^	21.8 ± 1.6^a^	33.8 ± 2.9^b^	^∗∗∗^	^∗^	^∗∗∗^
Met	64.6 ± 2.3^c^	41.7 ± 0.8^a^	39.8 ± 2.1^a^	50.9 ± 3.9^b^	^∗^	^∗^	^∗∗∗^
Val	145 ± 3.5^b^	121 ± 4.5^a^	109 ± 4.7^a^	146 ± 8.8^b^	ns	ns	^∗∗∗^
Ile	68.8 ± 2.1^b^	53.1 ± 2.1^a^	48.2 ± 1.7^a^	63.7 ± 4.2^b^	ns	ns	^∗∗∗^
Leu	129 ± 4.3^b^	98.1 ± 3.4^a^	88.8 ± 2.4^a^	117 ± 7.8^b^	ns	ns	^∗∗∗^
Phe	81.1 ± 1.2^c^	66.4 ± 1.1^b^	56.6 ± 2.1^a^	53.0 ± 3.0^a^	^∗∗∗^	^∗∗∗^
Total	3786 ± 38^b^	3419 ± 120^ab^	3085 ± 85^a^	4014 ± 370^b^	ns	ns	^∗^

*Different letters within a column indicate significant (P ≤ 0.05) differences between treatments. ^a^Analysis of variance: ns, not significant; ^∗^P ≤ 0.05; ^∗∗^P ≤ 0.01; ^∗∗∗^P ≤ 0.001. ^b^Nitrogen form.*

### Polyamines

**FIGURE 3:**
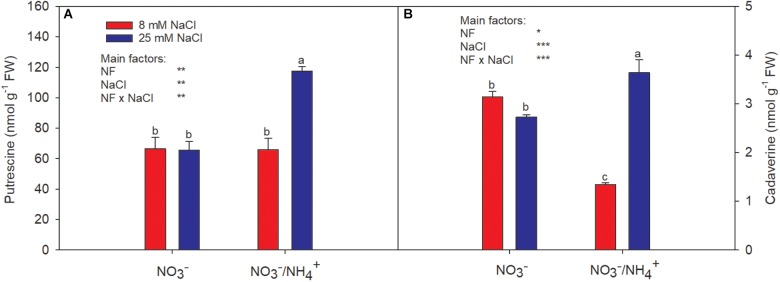
Effect of two different N forms (NF) (${\text{NO}}_{3}^{-}$ and ${\text{NO}}_{3}^{-}$ /${\text{NH}}_{4}^{+}$) and salinity on sweet pepper fruits: **(A)** putrescine and **(B)** cadaverine levels. Values with the same letter are not significantly different at *P* ≤ 0.05. Analysis of variance: ^∗^*P* ≤ 0.05; ^∗∗^*P* ≤ 0.01; ^∗∗∗^*P* ≤ 0.001.

Putrescine, cadaverine, and histamine were present in pepper fruit. However, histamine was scarcely detectable; therefore, only the data for putrescine and cadaverine are presented (Figure 3A,B). The putrescine levels in pepper fruit were not affected by salinity; however, the interactive effect of salinity and ${\text{NH}}_{4}^{+}$ caused a sharp increase (of 76%, compared to the control; Figure 3A). The cadaverine levels were significantly reduced by the supply of ${\text{NH}}_{4}^{+}$, from 3.14 to 1.34 nmol g^−1^ FW (Figure 3B), while the combined effect increased the cadaverine levels by 16%, compared to the control. The effects of salinity on polyamine biosynthesis have been studied in several plant species and the response seems to be dependent on the species, the plant system used, and/or the duration of exposure to salinity (Mutlu and Bozcuk, 2007). Houdusse et al. (2008), studying leaves of pepper plants, found that the effect of salinity depended on the N level. This agrees with Tiburcio et al. (1986), who indicated that dicotyledons submitted to osmotic stress had increased putrescine accumulation, but this was more dependent on the plant species considered (tomato) than on differences in N nutrition. In fruits, one of the main post-harvest effects attributed to polyamines is the preservation of their flesh firmness, which has been shown to be enhanced by salinity (Botella et al., 2000). Although ${\text{NH}}_{4}^{+}$ did not lead to increased concentrations of putrescine or cadaverine at the lower salinity level, at the higher level both polyamines were increased by the supply of ${\text{NH}}_{4}^{+}$. Beyond their contributions to stress tolerance in plants, polyamines (aliphatic amines) are involved in the regulation of differentiation of immune cells, inflammatory reactions, intestinal immunoallergic responses, diabetes, and food allergy prevention in children (Dandrifosse et al., 2000; Moinard et al., 2005). Therefore, their increased accumulation in plants in response to stress could enable benefits to be derived from the consumption of the edible parts of plants grown with the appropriate crop management, as the level of polyamines decreases with age in vital animal organs (Das and Kanungo, 1982).

## Conclusion

Irrigation with a nutrient solution in which ${\text{NO}}_{3}^{-}$ was partly replaced by ${\text{NH}}_{4}^{+}$ had important effects on the fruit quality of sweet pepper. There were changes in the color parameters and the mineral content was impaired, but at the same time the specific effect of a dramatic reduction in the Na concentration could be of paramount importance for specific dietary prescriptions. Additionally, the amino-acid profile was altered whilst the polyamines levels increased notably under salinity. Such responses should be taken into account in further studies that could include post-harvest quality and high salinity stress, to elucidate whether increasing polyamines levels can effectively counteract the deleterious effects of salinity on this crop. Moreover, crop management effects on polyamines accumulation should be taken into account with regard to dietary treatments of many ailments that affect the elderly.

## Data Availability

The datasets generated for this study are available on request to the corresponding author.

## Author Contributions

FdA, PL, MS-G, and EM conceived and supervised the whole study. FdA and MCP wrote the manuscript with inputs from all authors. MEP carried out the field experiment. MCP analyzed the plant material. MCP and JL-M performed the statistical analysis. All authors discussed the results and provided critical feedback on the manuscript.

## Conflict of Interest Statement

The authors declare that the research was conducted in the absence of any commercial or financial relationships that could be construed as a potential conflict of interest.
